# Analysis of the Relationship between Asthma and Coffee/Green Tea/Soda Intake

**DOI:** 10.3390/ijerph17207471

**Published:** 2020-10-14

**Authors:** Jee Hye Wee, Dae Myoung Yoo, Soo Hwan Byun, Chang Myeon Song, Hyo-Jeong Lee, Bumjung Park, Min Woo Park, Hyo Geun Choi

**Affiliations:** 1 Department of Otorhinolaryngology-Head & Neck Surgery, Hallym University Sacred Heart Hospital, Hallym University College of Medicine, Anyang 14068, Korea; weejh07@gmail.com (J.H.W.); hyobravo@gmail.com (H.-J.L.); bumjung426@gmail.com (B.P.); 2 Hallym Data Science Laboratory, Hallym University College of Medicine, Anyang 14068, Korea; ydm1285@naver.com; 3 Department of Oral & Maxillofacial Surgery, Dentistry, Hallym University Sacred Heart Hospital, Hallym University College of Medicine, Anyang 14068, Korea; purheit@daum.net; 4 Department of Otorhinolaryngology-Head & Neck Surgery, Hanyang University Seoul Hospital, Seoul 04763, Korea; cmsong@hanyang.ac.kr; 5 Department of Otorhinolaryngology-Head & Neck Surgery, Kangdong Sacred Heart Hospital, Seoul 05355, Korea; subintern@naver.com

**Keywords:** asthma, coffee, tea, sugar-sweetened beverages, caffeine, population surveillance

## Abstract

This study aimed to evaluate the association between asthma and the intake of coffee/green tea/soda. We used Health Examinee data from the Korean Genome and Epidemiology Study (2004–2016). The participants (*n* = 3146 with asthma; *n* = 158,902 non-asthma) were asked about the frequency and amount of their coffee/green tea/soda intake. Multiple logistic regression analyses were used to calculate the adjusted odds ratios (aORs) with 95% confidence intervals (CIs) for asthma according to the frequency and amount of coffee/green tea/soda intake. Compared to the group consuming no coffee, the aORs for asthma were 0.82 (95% CI = 0.73–0.93, *p* = 0.002) in the group consuming coffee 1–2 times/day and 0.87 (95% CI = 0.78–0.97, *p* = 0.011) in the group consuming coffee in quantities of 1 cup, respectively. However, the frequency and amount of green tea and soda consumption were not significantly associated with asthma after adjusting for coffee consumption (all *p* > 0.05). These findings were consistent in the female subgroup (1–2 times/day: aOR = 0.76, 95% CI = 0.66–0.87, *p* < 0.001, and 1 cup each time: aOR = 0.79, 95% CI = 0.70–0.90, *p* < 0.001) but not in the male subgroup. Drinking 1 cup of coffee each time and 1–2 times per day may have protective effects against asthma in a Korean population. However, the associations between asthma and green tea/soda cannot be clearly established.

## 1. Introduction

Asthma is the most common chronic respiratory disease and is characterized by allergic inflammation and constriction of the airways, with acute attacks. From 1990 to 2015, the prevalence of asthma increased by 12.6% (9.0–16.4) to 358.2 million individuals (323.1–393.5 million) [1]. The annual incidence of asthma increased from 3.63 per 1000 person-years in 2004 to 6.07 per 1000 person-years in 2008 [2], and the cost associated with asthma, representing the sum of all direct, indirect, and intangible costs, was $4.11 billion [3] in Korea.

Coffee, green tea, and soda are the most commonly consumed beverages worldwide. Coffee intake is rapidly increasing in the Korean population. Among the various types of tea, green tea is widely consumed in East Asia, including China, Japan, and Korea. Many studies have been reported on the health effects of these beverages [4,5,6,7]. A study from the Korean National Health and Nutrition Examination Survey reported that the frequent consumption of green tea (≥3 cups/week) or coffee (≥2 cups/day) was associated with a reduced prevalence of depression (odds ratio (OR) = 0.79, 95% confidence interval (CI) = 0.63–0.99, OR = 0.68, 95% CI = 0.55–0.85) in Korean adults [4]. A study from the National Health and Nutrition Examination Survey showed that coffee consumption (≥6 cups daily) was associated with the frequency of hyperuricemia (OR = 0.57, 95% CI = 0.35–0.94), but tea consumption (≥6 cups daily) was not (OR = 1.00, 95% CI = 0.65–1.53) in adults in the U.S [5]. In a cross-sectional study of Australian women, drinking 1–2 cups of coffee or tea per day was associated with a 17–26% increase in physical activity [6]. A meta-analysis found that people who drank 4 or more cups of coffee daily had a risk of colorectal cancer that was 24% lower than that of non-coffee drinkers [7].

A recent systematic review showed inconsistent results from three studies assessing the association between coffee consumption and asthma [8]. In a study using the Italian National Health Survey, the relative risks of bronchial asthma were 0.79 (95% CI = 0.69–0.91) for those who consumed two cups per day and 0.78 (95% CI = 0.69–0.88) for those who consumed three or more cups per day compared with subjects who did not drink coffee [9]. Similarly, a U.S. study using data from the National Health and Nutritional Examination Survey II also found that subjects who drank coffee on a regular basis had a 29% reduction in the odds of having current asthma symptoms (0.71, 95% CI = 0.55–0.93) compared to non-coffee drinkers [10]. In contrast, in the French National Health Survey, there was no relation between the level of coffee or tea consumption and asthma prevalence [11].

However, growing evidence suggests that the consumption of sugar-sweetened beverages, soda, and fructose during pregnancy/childhood/adulthood may lead to asthma development. In a meta-analysis [12], soft drink consumption was associated with significantly increased odds of asthma in both adults (OR = 1.37, 95% CI = 1.23–1.52) and children (OR = 1.14, 95% CI = 1.06–1.21). Prenatal maternal consumption of soft drinks was significantly associated (OR = 1.11, 95% CI = 1.00–1.23) with asthma in children.

We hypothesized that the intake of coffee and green tea might have a protective effect on asthma due to the effects of caffeine as a bronchodilator and the modulation of allergic inflammation, and the soda intake would have a complex effect on asthma due to the effects of both caffeine and sugar. The aim of this study was to evaluate the association between asthma and the intake of coffee/green tea/soda in an adult Korean population.

## 2. Materials and Methods

### 2.1. Study Population and Data Collection

The ethics committee of Hallym University (2019-02-020) approved the use of these data. The requirement for written informed consent was waived by the Institutional Review Board. This cross-sectional study used the data of the Health Examinee (HEXA) population-based cohort from the Korean Genome and Epidemiology Study (KoGES), which is a consortium project consisting of six prospective cohort studies. A detailed description of these data was provided in a previous study [13]. The urban community based KoGES_HEXA study involves volunteers aged ≥ 40 years who visited the involved institutions, which are mainly general hospitals in the metropolitan areas and major Korean cities. The baseline recruitment was conducted in 39 sites from 2004 to 2013, and the follow-up data were obtained from 2012 to 2016. We obtained the previous history of asthma and the frequency and amount of consumption of drinks from the baseline data. In addition, patients who reported having doctor-diagnosed asthma during the follow-up period were included (*n* = 369).

### 2.2. Participant Selection

**Figure 1:**
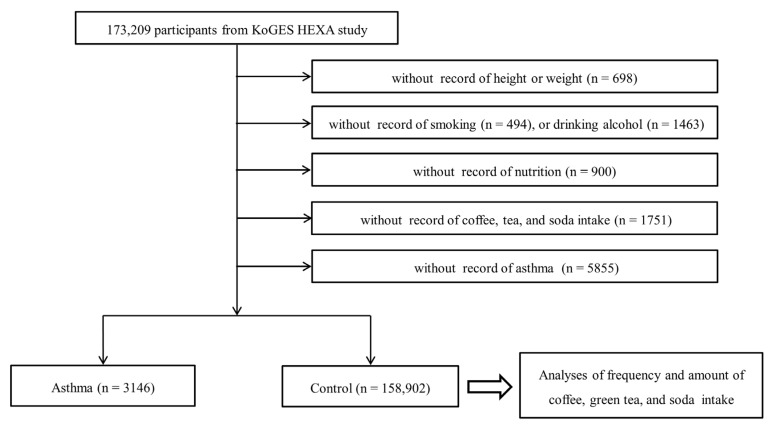
A schematic illustration of the participant selection process used in the present study. In total, 173,209 participants were included.

Among 173,209 participants, we excluded participants who lacked records of height or weight (*n* = 698), smoking history (*n* = 494), alcohol consumption habits (*n* = 1463), nutrition records (*n* = 900), coffee/green tea/soda intake (*n* = 1751), and asthma history (*n* = 5855). Many participants were excluded due to the lack of information about their asthma history because asthma was not surveyed in 2004. Finally, 3146 asthma participants and 158,902 non-asthma participants were selected (Figure 1). Then, we analyzed the histories of the frequency of coffee/green tea/soda intake (primary object). We also analyzed the amount of coffee/green tea/soda intake (secondary object).

### 2.3. Survey

The participants were asked about their sociodemographic status, lifestyle (e.g., smoking and alcohol drinking), and disease histories by trained interviewers using a questionnaire. The participants were defined as asthma patients if they were previously diagnosed by a medical doctor. Body mass index (BMI) was calculated in kg/m^2^ using the health checkup data and was categorized as <18.5 (underweight), ≥18.5 to <23 (normal), ≥23 to <25 (overweight), and ≥25 (obese) based on the Asia-Pacific criteria following the Western Pacific Regional Office (WPRO) 2000 [14]. Participants were categorized according to their smoking history as nonsmokers (<100 cigarettes in their lifetime), past smokers (quit more than one year prior), and current smokers. The participants were categorized according to their alcohol consumption habits as nondrinkers (<12 times a year and <1 cup each time), past drinkers, and current drinkers. Their nutritional intake (total calories (kcal/day), protein (g/day), fat (g/day), and carbohydrate (g/day)) was surveyed by a food-frequency questionnaire that was validated in a previous study [15]. The household income groups were unknown, low income (<~$2000 per month), middle income (~$2000–3999 per month), and high income (~≥$4000 per month).

The frequency of coffee/green tea/soda consumption was categorized as none, once a month, 2–3 times a month, 1–2 times a week, 3–4 times a week, 5–6 times a week, 1–2 times a day, 3–4 times a day, and more than 5 times a day. Because many participants were included in the 1–2 times a day group, we condensed the categories into the following 4 groups: none, 1 time a month through 6 times a week, 1–2 times a day, and ≥3 times a day.

The amount of coffee/green tea/soda was categorized as none, 1/2 cup each time, 1 cup each time, and 2 cups each time.

### 2.4. Statistical Analysis

Chi-square tests were used to compare the sex, BMI category, income, smoking status, alcohol consumption and frequency, and amount of coffee/green tea/soda consumption. Independent T-tests were used to compare age and nutritional intake including total calories, protein, fat, and carbohydrates.

To analyze the OR of asthma based on coffee, green tea, and soda intake (frequency/amount), a crude model, model 1 (adjusted for age, sex, BMI category, smoking status, alcohol consumption, and nutritional intake (total calories, protein, fat, and carbohydrate)), and model 2 (model 1 plus coffee, green tea, and soda consumption) were used. In the subgroup analyses according to age, the cutoff point was determined by the median age (<53 years old and ≥53 years old). The potential interactions were formally tested by including interaction terms.

Two-tailed analyses were conducted, and *p*-values less than 0.05 were considered to indicate significance. The results were statistically analyzed using SPSS v. 24.0 (IBM, Armonk, NY, USA).

## 3. Results

Age, sex, obesity, income, smoking status, alcohol consumption, intake of total calories/protein/fat, frequency and amount of coffee consumption, and frequency of green tea and soda consumption differed between participants with asthma and non-asthma (all *p* < 0.05, Table 1). Thus, these variables were adjusted for in the logistic regression analysis of the relations between coffee/green tea/soda and asthma.

According to model 2, the ORs for asthma were 0.98 (95% CI = 0.85–1.12, *p* = 0.743), 0.82 (95% CI = 0.73–0.93, *p* = 0.002), and 0.86 (95% CI = 0.73–1.02, *p* = 0.086) in the groups consuming coffee less than 1 time/day, 1–2 times/day, and more than 3 times/day, respectively (Table 2). According to model 2, the ORs for asthma were 1.06 (95% CI = 0.87–1.29, *p* = 0.572), 0.87 (95% CI = 0.78–0.97, *p* = 0.011), 0.87 (95% CI = 0.78–0.97, *p* = 0.011) in the groups consuming 1/2 cup, 1 cup, and 2 cups of coffee each time (Table 3). However, the frequency and amount of green tea and soda consumption were not significantly associated with asthma in model 2 (all *p* > 0.05).

**Figure 2:**
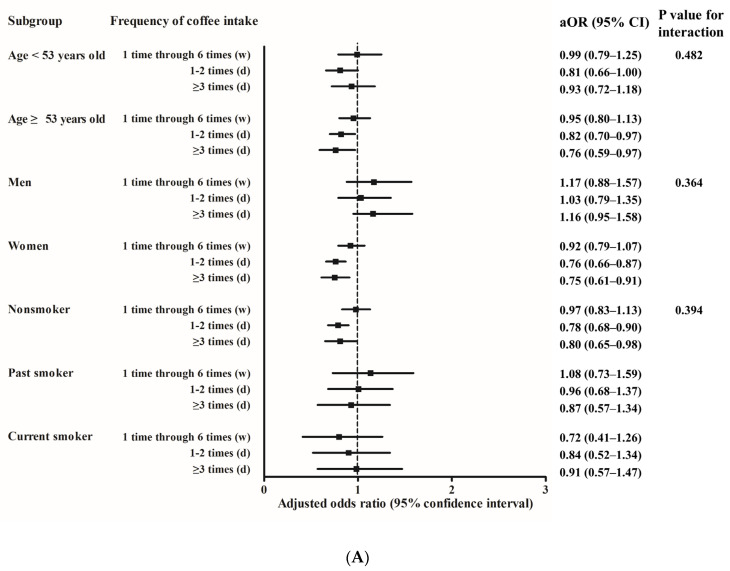

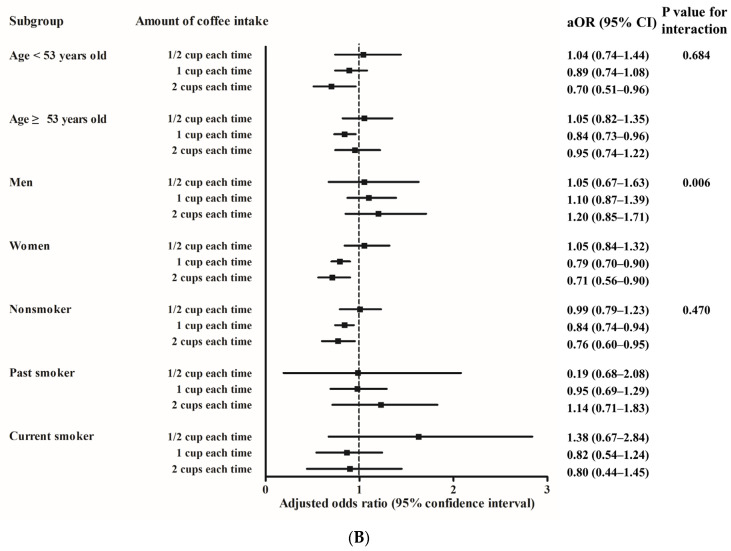
Subgroup analyses according to age, sex, and smoking status. Adjusted odds ratios (95% confidence interval) of coffee intake ((**A**) frequency, (**B**) amount) for asthma based on multiple logistic regression analyses adjusted for age, sex, BMI category, income, smoking status, alcohol consumption, nutritional intake (total calories, protein, fat, and carbohydrate), and frequency or amount of green tea and soda drink intake.

Subgroup analyses according to age, sex, and smoking status were conducted. In model 2, asthma was negatively associated with frequent coffee consumption among the women (1–2 times/day: aOR = 0.76, 95% CI = 0.66–0.87, *p* < 0.001, and ≥3 times/day: aOR = 0.75, 95% CI = 0.61–0.91, *p* = 0.005), participants aged over 53 years (1–2 times/day: aOR = 0.82, 95% CI = 0.70–0.97, *p* = 0.017, and ≥3 times/day: aOR = 0.76, 95% CI = 0.59–0.97, *p* = 0.027), and nonsmokers (1–2 times/day: aOR = 0.78, 95% CI = 0.68–0.90, *p* < 0.001, and ≥3 times/day: aOR = 0.80, 95% CI = 0.65–0.98, *p* = 0.029) (Figure 2A, Supplementary Table S1). Compared to the participants who did not drink coffee, the ORs for asthma were significantly lower among the women who drank 1 cup (0.79, 95% CI = 0.70–0.90, *p* < 0.001) and 2 cups (0.71, 95% CI = 0.56–0.90, *p* = 0.005) of coffee each time, the participants aged less than 53 years who drank 2 cups of coffee each time (0.70, 95% CI = 0.51–0.96, *p* = 0.027), the participants older than 53 years who drank 1 cup of coffee each time (0.84, 95% CI = 0.71–0.96, *p* = 0.010), and the nonsmokers who drank 1 cup (0.84, 95% CI = 0.74–0.94, *p* = 0.004) and 2 cups (0.76, 95% CI = 0.60–0.95, *p* = 0.019) of coffee each time (Figure 2B, Supplementary Table S2). However, the frequency and amount of green tea and soda consumption was not significantly negatively associated with asthma in any age group, sex group, or smoking status group (Supplementary Tables S3–S6).

## 4. Discussion

The present study showed that coffee consumption was associated with a lower prevalence of asthma, but green tea and soda were not. These findings were consistent in the female subgroup but not in the male subgroup.

The effects of methylxanthines could contribute to the inverse association of coffee and asthma. Caffeine (1,3,7-trimethylxanthine), theobromine (3,7-dimethylxanthine), and theophylline (1,3-dimethylxanthine) belong to the compound group of the methylxanthines and are some of the main components in coffee [16]. First, methylxanthines have bronchodilator effects. The mechanism of action of methylxanthine has been described [17] and involves the mobilization of intracellular calcium, inhibition of phosphodiesterases (PDEs), modulation of gamma-Aminobutyric acid type A (GABA_A_) receptors, and antagonism of adenosine receptors. Muscle contraction is regulated by intracellular levels of cyclic adenosine monophosphate (cAMP), which is synthesized by adenylate cyclases and hydrolyzed by PDEs. Therefore, the inhibition of PDEs by methylxanthines relaxes the smooth muscle in the airway. A previous study showed that significant improvement from baseline values on pulmonary function tests was noted after the ingestion of either caffeine or theophylline [18]. Second, adenosine receptor antagonism by methylxanthines may modulate allergic inflammation. This is because adenosine is a biological mediator with the capacity to produce inflammatory effects in tissues [19]. Finally, previous studies have proposed that caffeine has antioxidant and prooxidant properties and therefore protects humans against conditions associated with oxidative stress [20,21]. There is evidence for the presence of oxidative stress in asthma, which is closely related to disease-related airflow obstruction, airway hyperreactivity and remodeling [22]. Caffeine has antioxidant effects and can therefore play an important role in preventing asthma through the scavenging of free radicals.

Asthma was also inversely associated with drinking green tea 1–2 times/day (OR = 0.86, 95% CI = 0.78–0.93, *p* < 0.001 and OR = 0.87, 95% CI = 0.80–0.95, *p* = 0.002) in the crude model and model 1. The inverse association between consuming soda 1–2 times/day and asthma was only observed in the crude model (OR = 0.82, 95% CI = 0.73–0.93, *p* = 0.001). However, after adjusting for the frequency of coffee intake, the association between asthma and green tea/soda disappeared (*p* = 0.790 for green tea, *p* = 0.344 for soda drinks). These results may be explained by the following: (1) the different caffeine contents of these beverages, (2) the other components of coffee, and (3) the dominant effects of drinking coffee. An eight-ounce (240 mL) cup of coffee contains 80 to 200 mg of caffeine [23,24]. Green tea and soda usually contain approximately 25 to 40 mg of caffeine per 8-ounce serving [23,24], which is approximately one-quarter of the amount found in a typical cup of coffee. In a Cochrane review [25], 5 mg/kg body weight of caffeine achieved a peak bronchodilator effect within 2 h and lasted for six hours. Previous studies showed a dose-response relationship between coffee consumption and asthma prevalence [9,10]. Therefore, it can be assumed that only coffee with a large amount of caffeine has a protective effect against asthma. In addition, components of coffee other than caffeine may be responsible for the effect on asthma reduction. Chlorogenic acid in coffee has been reported to have antioxidant and anti-inflammatory effects [26]. Moreover, it can be assumed that drinking coffee has a dominant effect. The dominant effect of drinking coffee might be able to mask the effects of drinking green tea or soda. Collinearity may exist because the subjects who frequently drink green tea or soda may also frequently consume coffee.

Moreover, soda drinks usually contain sugar as well as caffeine. Previous studies have shown that the consumption of sugar-sweetened beverages was associated with greater odds of having asthma. Among nonobese U.S. adults (BMI < 30 kg/m^2^), the odds of having current asthma in the subjects who consumed sugar-sweetened beverages ≥ 2 times/day were significantly higher (adjusted OR = 1.66, 95% CI = 1.39–1.99) than those in the subjects who did not consume sugar-sweetened beverages [27]. In a study using data from the Framingham Offspring Cohort [28], regular (5–7 times/week) consumers of high-fructose corn syrup-sweetened soda had a 49% higher asthma risk (hazard ratio = 1.49, 95% CI = 1.10–2.02, *p* = 0.011) as compared with never/seldom consumers. However, there was no association between diet soda intake and asthma (hazard ratio = 0.93, 95% CI = 0.72–1.20, *p* = 0.585). The association between asthma and soda cannot be explained because the present study did not distinguish among different types of soda drinks (caffeine/no caffeine, sweetened/sugar free etc.).

In the subgroup analysis, coffee consumption was inversely associated with asthma in women but not in men. This difference may originate from the difference in response to caffeine by sex. Caffeine is metabolized by the cytochrome P450 1A2 (CYP1A2) enzyme. Men metabolize caffeine at a higher rate than women because CYP1A2 has higher activity levels in men than in women [29]. Estradiol inhibits CYP1A2 activity, resulting in reduced caffeine clearance [30,31]. Furthermore, a previous study reported that higher caffeine intake was associated with an increased free estradiol concentration among Asian women, whereas it was inversely associated among Caucasian women [32]. An alternative explanation is that these sex differences are related to differences in caffeine consumption patterns (Supplementary Table S7). Sex differences in patterns of caffeine consumption have also been shown in other studies [33]. In addition, compared to the analysis of the female participants, the analysis of the male participants may lack statistical power. In this study, the number of male participants (*n* = 55,559) was smaller than that of female participants (*n* = 106,489).

This study has some limitations. First, we could not account for the total amount of coffee/green tea/soda consumed per day because participants were asked about the frequency and amount separately for each category. As these variables were assessed using bins (such as >5 time a day), we could not simply multiply the frequency and by the amount of the drinks. Second, we could not assess the actual caffeine and other methylxanthine (theophylline or theobromine) intake. There was no information about brewing time and temperature, grade, and varietal characteristics of the coffee and whether or not decaffeinated coffee was consumed. The different types of sodas consumed (caffeine/no caffeine, sweetened/sugar free, etc.) were unknown. However, an epidemiological study evaluated the association between caffeine intake and health outcomes using the amount and frequency of coffee consumption [34], and decaffeinated coffee was still not very commonly consumed in Korea [35]. Third, the association between the amount of coffee consumed and asthma was not dose-dependent, although there was a dose-response relationship between coffee consumption and asthma in the subgroup of women < 53 years old. This might be because the majority of participants (78.6% of males, 71.8% of females) stated that they consumed 1 cup of coffee each time (Supplementary Table S7). Fourth, although we adjusted for several potential confounders, we could not exclude the possibility of residual confounding by other unmeasured variables, such as physical activity, environmental factors (e.g., passive smoke exposure and industrial exposure), and other foods. Finally, given the nature of a cross-sectional study, we cannot conclude whether coffee intake is a determinant of asthma or whether the reverse is true. Although the total number of developed asthma patients was very small (*n* = 369, 11.4%), we analyzed the incidence of asthma during the follow-up period according to the frequency and amount of coffee/green tea/soda drink intake. It showed that the incidence of developed asthma was significantly different according to the frequency and amount of each drink, but it was not in a dose-dependent manner (Supplementary Table S8). Future studies should employ a prospective design to further evaluate the effect and the mechanism of action by which coffee consumption affects asthma.

Despite these limitations, this study is meaningful because the results are representative of those in the general population after adjusting for several covariates, including smoking status and alcohol consumption. Cigarette smoking increases caffeine clearance by inducing CYP1A2 activity [36], and smokers have been found to have lower plasma levels of caffeine than nonsmokers at the same level of consumption [37]. Furthermore, we analyzed the association between asthma and coffee/green tea/soda consumption using the model not adjusted for BMI and each nutritional intake (Supplementary Table S9), because such a model may over adjust the diet/beverage–asthma association due to the likely mediating role of BMI (especially given that the analyses were already adjusted for the total calories). In addition, because there is a potential correlation between soda and sugar intake, a model adjusting carbohydrate may over adjust the diet/soda–asthma association. However, the results were consistent with those obtained after adjusting for BMI and nutritional intake (total calories, protein, fat, and carbohydrate).

## 5. Conclusions

Drinking 1 cup of coffee each time and drinking coffee 1–2 times per day may have protective effects against asthma in the Korean population. The inverse associations between coffee consumption and asthma were particularly observed in women. However, we cannot conclude whether there are associations between asthma and green tea/soda.

## Figures and Tables

**Table 1:** General characteristics of the participants.

Characteristics	Total Participants		*p*-Value
	Asthma	Non-Asthma	
Age (mean, SD, y)	55.9 (8.7)	53.2 (8.4)	<0.001 ^1^
Sex (*n*, %)			<0.001 ^1^
Men	946 (30.1)	54,613 (34.4)	
Women	2200 (69.9)	104,289 (65.6)	
Obesity (*n*, %)			<0.001 ^1^
Underweight (BMI < 18.5 kg/m^2^)	63 (2.0)	2838 (1.8)	
Normal (18.5 kg/m^2^ ≤ BMI < 23 kg/m^2^)	1023 (32.5)	59,608 (37.5)	
Overweight (23 kg/m^2^ ≤ BMI < 25 kg/m^2^)	846 (26.9)	44,162 (27.8)	
Obese (25 kg/m^2^ ≤ BMI)	1214 (38.6)	52,294 (32.9)	
Income (*n*, %)			<0.001 ^1^
Missing, no response	467 (14.8)	20,194 (12.7)	
Lowest	1121 (35.6)	44,817 (28.2)	
Middle	993 (37.4)	59,491 (37.4)	
Highest	565 (21.6)	34,400 (21.6)	
Smoking status (*n*, %)			<0.001 ^1^
Nonsmoker	2337 (74.3)	115,848 (72.9)	
Past smoker	505 (16.1)	23,256 (14.6)	
Current smoker	304 (9.7)	19,798 (12.5)	
Alcohol consumption (*n*, %)			<0.001 ^1^
Nondrinker	1794 (57.0)	80,746 (50.8)	
Past drinker	175 (5.6)	6070 (3.8)	
Current drinker	1177 (37.4)	72,086 (45.4)	
Nutritional intake (mean, SD)			
Total calories (kcal/d)	1722.4 (574.2)	1755.3 (581.9)	0.002 ^1^
Protein (g/d)	57.8 (25.4)	59.7 (26.8)	<0.001 ^1^
Fat (g/d)	26.5 (17.3)	28.1 (18.4)	<0.001 ^1^
Carbohydrate (g/d)	309.1 (96.4)	311.8 (95.2)	0.114
Frequency of coffee (*n*, %)			<0.001 ^1^
None	643 (20.4)	26,464 (16.7)	
1 time (m) through 6 times (w)	779 (24.8)	33,799 (21.3)	
1–2 times (d)	1201 (38.2)	67,273 (42.3)	
≥3 times (d)	523 (16.6)	31,366 (19.7)	
Amount of coffee (*n*, %)			<0.001 ^1^
None	643 (20.4)	26,464 (16.7)	
1/2 cup each time	142 (4.5)	6118 (3.9)	
1 cup each time	2213 (70.3)	117,884 (74.2)	
2 cups each time	148 (4.7)	8436 (5.3)	
Frequency of green tea (*n*, %)			<0.001 ^1^
None	1421 (45.2)	68,171 (42.9)	
1 time (m) through 6 times (w)	589 (18.7)	25,616 (16.1)	
1–2 times (d)	803 (25.5)	45,069 (28.4)	
≥3 times (d)	333 (10.6)	20,046 (12.6)	
Amount of green tea (*n*, %)			0.057
None	1421 (45.2)	68,171 (42.9)	
1/2 cup each time	504 (16.0)	26,672 (16.8)	
1 cup each time	1168 (37.1)	61,640 (38.8)	
2 cups each time	53 (1.7)	2419 (1.5)	
Frequency of soda drinks (*n*, %)			0.004 ^1^
None	1290 (41.0)	63,960 (40.3)	
1 time (m) through 6 times (w)	1399 (45.5)	68,108 (42.9)	
1–2 times (d)	350 (11.1)	21,097 (13.3)	
≥3 times (d)	107 (3.4)	5737 (3.6)	
Amount of soda drinks (*n*, %)			0.866
None	1290 (41.0)	63,960 (40.3)	
1/2 cup each time	107 (3.4)	5472 (3.4)	
1 cup each time	1667 (53.0)	85,257 (53.7)	
2 cups each time	82 (2.6)	4213 (2.7)	

^1^ Independent T-test or Chi-square test. Significance at *p* < 0.05. SD = standard deviation; d = day; w = week; m = month; BMI = body mass index.

**Table 2:** Crude and adjusted odds ratios (95% confidence interval) for asthma by coffee, green tea, and soda drink intake (frequency).

Characteristics	Odds Ratios for Asthma					
	Crude	*p*-Value	Model 1 ^2^	*p*-Value	Model 2 ^3^	*p*-Value
Coffee						
None	1.00		1.00		1.00	
1 time (m) through 6 times (w)	0.95 (0.85–1.05)	0.328	1.03 (0.93–1.15)	0.565	0.98 (0.85–1.12)	0.743
1–2 times (d)	0.74 (0.67–0.81)	<0.001 ^1^	0.81 (0.74–0.90)	<0.001 ^1^	0.82 (0.73–0.93)	0.002 ^1^
≥3 times (d)	0.69 (0.61–0.77)	<0.001 ^1^	0.85 (0.75–0.96)	0.010 ^1^	0.86 (0.73–1.02)	0.086
Green tea						
None	1.00		1.00		1.00	
1 time (m) through 6 times (w)	1.10 (1.00–1.22)	0.048 ^1^	1.11 (1.01–1.23)	0.031 ^1^	1.06 (0.94–1.21)	0.331
1–2 times (d)	0.86 (0.78–0.93)	<0.001 ^1^	0.87 (0.80–0.95)	0.002 ^1^	0.98 (0.88–1.11)	0.790
≥3 times (d)	0.80 (0.71–0.90)	<0.001 ^1^	0.91 (0.80–1.03)	0.149	0.98 (0.81–1.17)	0.790
Soda drinks						
None	1.00		1.00		1.00	
1 time (m) through 6 times (w)	1.02 (0.94–1.10)	0.639	1.09 (1.01–1.18)	0.022 ^1^	1.07 (0.99–1.15)	0.106
1–2 times (d)	0.82 (0.73–0.93)	0.001 ^1^	0.92 (0.81–1.04)	0.185	0.94 (0.83–1.06)	0.344
≥3 times (d)	0.93 (0.76–1.13)	0.441	1.07 (0.87–1.31)	0.528	1.07 (0.87–1.31)	0.563

^1^ Logistic regression model, Significance at *p* < 0.05. ^2^ Model 1 was adjusted for age, sex, BMI category, income, smoking status, alcohol consumption, and nutritional intake (total calories, protein, fat, and carbohydrate). ^3^ Model 2 was adjusted for model 1 plus frequency of coffee, green tea, and soda drink consumption. d = day; w = week; m = month.

**Table 3:** Crude and adjusted odds ratios (95% confidence interval) for asthma by coffee, green tea, and soda drink intake (amount).

Characteristics	Odds Ratios for Asthma					
	Crude	*p*-Value	Model 1 ^2^	*p*-Value	Model 2 ^3^	*p*-Value
Coffee						
None	1.00		1.00		1.00	
1/2 cup each time	0.96 (0.80–1.15)	0.626	1.06 (0.88–1.28)	0.515	1.06 (0.87–1.29)	0.572
1 cup each time	0.77 (0.71–0.84)	<0.001 ^1^	0.88 (0.80–0.96)	0.005 ^1^	0.87 (0.78–0.97)	0.011 ^1^
2 cups each time	0.72 (0.60–0.87)	<0.001 ^1^	0.86 (0.71–1.03)	0.105	0.84 (0.69–1.02)	0.080
Green tea						
None	1.00		1.00		1.00	
1/2 cup each time	0.91 (0.82–1.00)	0.061	0.99 (0.89–1.10)	0.806	1.03 (0.92–1.15)	0.601
1 cup each time	0.91 (0.84–0.98)	0.017 ^1^	0.94 (0.86–1.01)	0.111	1.00 (0.91–1.10)	0.962
2 cups each time	1.05 (0.80–1.39)	0.725	1.03 (0.78–1.36)	0.826	1.11 (0.82–1.47)	0.488
Soda drinks						
None	1.00		1.00		1.00	
1/2 cup each time	0.97 (0.79–1.18)	0.761	1.04 (0.85–1.27)	0.727	0.98 (0.79–1.20)	0.834
1 cup each time	0.97 (0.90–1.04)	0.407	1.06 (0.98–1.14)	0.152	1.06 (0.98–1.14)	0.130
2 cups each time	0.97 (0.77–1.21)	0.757	1.13 (0.90–1.42)	0.280	1.13 (0.90–1.42)	0.296

^1^ Logistic regression model, Significance at *p* < 0.05. ^2^ Model 1 was adjusted for age, sex, BMI category, income, smoking status, alcohol consumption, and nutritional intake (total calories, protein, fat, and carbohydrate). ^3^ Model 2 was adjusted for model 1 plus amount of coffee, green tea, and soda drink consumption.

## Supplementary Materials

The following are available online at https://www.mdpi.com/1660-4601/17/20/7471/s1, Table S1: Crude and adjusted odds ratios (95% confidence interval) for asthma by coffee intake (frequency) according to age, sex, and smoking status, Table S2: Crude and adjusted odds ratios (95% confidence interval) for asthma by coffee intake (amount) according to age, sex, and smoking status, Table S3: Crude and adjusted odds ratios (95% confidence interval) for asthma by green tea intake (frequency) according to age, sex, and smoking status, Table S4: Crude and adjusted odds ratios (95% confidence interval) for asthma by green tea intake (amount) according to age, sex, and smoking status, Table S5: Crude and adjusted odds ratios (95% confidence interval) for asthma by soda drink intake (frequency) according to age, sex, and smoking status, Table S6: Crude and adjusted odds ratios (95% confidence interval) for asthma by soda drink intake (amount) according to age, sex, and smoking status, Table S7: Frequency of coffee/green tea/soda drink intake according to sex, Table S8: Incidence of developed asthma during the follow-up period according to the frequency and amount of coffee/green tea/soda drink intake, Table S9: Adjusted odds ratios (95% confidence interval) for asthma by coffee, green tea, and soda drink intake.
