# Supplementary material for: Analysis of the Relationship between Asthma and Coffee/Green Tea/Soda Intake
Source: Int J Environ Res Public Health. 2020 Oct 14;17(20):7471. doi: 10.3390/ijerph17207471 (PMC7602133; doi:10.3390/ijerph17207471)
Supplement: Supplementary file 1 [file ijerph-17-07471-s001.pdf]

Table S1. Crude and adjusted odds ratios (95% confidence interval) for asthma by coffee intake (frequency) according to age, sex, and smoking status.

Characteristics	Odds ratios for asthma					
	Crude	P-value	Model 1[2]	P-value	Model 2[3]	P-value
Age < 53 years old (n = 80,079)						
None	1.00		1.00		1.00	
1 time (m) through 6 times (w)	1.01 (0.83-1.22)	0.951	1.00 (0.82-1.21)	0.976	0.99 (0.79-1.25)	0.965
1-2 times (d)	0.78 (0.55-0.93)	0.006[1]	0.77 (0.64-0.92)	0.005[1]	0.81 (0.66-1.00)	0.053
≥ 3 times (d)	0.78 (0.64-0.95)	0.013[1]	0.82 (0.66-1.00)	0.050	0.93 (0.72-1.18)	0.540
Age ≥ 53 years old (n = 81,969)						
None	1.00		1.00		1.00	
1 time (m) through 6 times (w)	0.99 (0.87-1.13)	0.901	1.04 (0.92-1.19)	0.500	0.95 (0.80-1.13)	0.603
1-2 times (d)	0.78 (0.70-0.88)	<0.001[1]	0.83 (0.73-0.93)	0.002[1]	0.82 (0.70-0.97)	0.017[1]
≥ 3 times (d)	0.77 (0.66-0.89)	0.001[1]	0.85 (0.72-1.00)	0.056	0.76 (0.59-0.97)	0.027[1]
Men (n = 55,559)						
None	1.00		1.00		1.00	
1 time (m) through 6 times (w)	1.32 (1.05-1.65)	0.015[1]	1.45 (1.16-1.82)	0.001[1]	1.17 (0.88-1.57)	0.275
1-2 times (d)	0.98 (0.79-1.21)	0.842	1.08 (0.88-1.34)	0.456	1.03 (0.79-1.35)	0.821
≥ 3 times (d)	0.89 (0.72-1.11)	0.317	1.11 (0.88-1.40)	0.366	1.16 (0.95-1.58)	0.332
Women (n = 106,489)						
None	1.00		1.00		1.00	
1 time (m) through 6 times (w)	0.87 (0.77-0.98)	0.021[1]	0.92 (0.81-1.04)	0.191	0.92 (0.79-1.07)	0.288
1-2 times (d)	0.68 (0.61-0.76)	<0.001[1]	0.73 (0.66-0.82)	<0.001[1]	0.76 (0.66-0.87)	<0.001[1]
≥ 3 times (d)	0.68 (0.58-0.78)	<0.001[1]	0.76 (0.65-0.89)	0.001	0.75 (0.61-0.91)	0.005[1]
Nonsmoker (n = 118,185)						
None	1.00		1.00		1.00	
1 time (m) through 6 times (w)	0.93 (0.83-1.05)	0.248	0.99 (0.88-1.12)	0.920	0.97 (0.83-1.13)	0.691
1-2 times (d)	0.71 (0.64-0.79)	<0.001[1]	0.77 (0.69-0.86)	<0.001[1]	0.78 (0.68-0.90)	<0.001[1]

≥ 3 times (d)	0.70 (0.60-0.81)	<0.001[1]	0.82 (0.71-0.96)	0.012[1]	0.80 (0.65-0.98)	0.029[1]
Past smoker (n = 23,761)						
None	1.00		1.00		1.00	
1 time (m) through 6 times (w)	1.22 (0.90-1.66)	0.174	1.30 (0.95-1.77)	0.095	1.08 (0.73-1.59)	0.697
1-2 times (d)	1.03 (078-1.36)	0.846	1.07 (0.81-1.42)	0.632	0.96 (0.68-1.37)	0.839
≥ 3 times (d)	0.88 (0.64-1.20)	0.416	0.98 (0.71-1.34)	0.881	0.87 (0.57-1.34)	0.532
Current smoker (n = 20,102)						
None	1.00		1.00		1.00	
1 time (m) through 6 times (w)	074 (0.48-1.15)	0.184	0.87 (0.56-1.35)	0.531	0.72 (0.41-1.26)	0.248
1-2 times (d)	0.58 (0.39-0.86)	0.006[1]	0.67 (0.45-0.99)	0.047[1]	0.84 (0.52-1.34)	0.461
≥ 3 times (d)	0.58 (0.40-0.86)	0.006[1]	0.73 (0.49-1.09)	0.122	0.91 (0.57-1.47)	0.712

[1] Logistic regression model, Significance at $P < 0.05$

[2] Model 1 was adjusted for age, sex, BMI category, income, smoking status, alcohol consumption, and nutritional intake (total calories, protein, fat, and carbohydrate).

[3] Model 2 was adjusted for model 1 plus frequency of green tea and soda drink intake.

Table S2. Crude and adjusted odds ratios (95% confidence interval) for asthma by coffee intake (amount) according to age, sex, and smoking status.

Characteristics	Odds ratios for asthma					
	Crude	P-value	Model 1[2]	P-value	Model 2[3]	P-value
Age < 53 years old (n = 80,079)						
None	1.00		1.00		1.00	
1/2 cup each time	0.98 (0.72-1.34)	0.915	0.96 (0.70-1.32)	0.816	1.04 (0.74-1.44)	0.828
1 cup each time	0.84 (0.71-0.99)	0.035[1]	0.85 (0.71-1.00)	0.054	0.89 (0.74-1.08)	0.246
2 cups each time	0.68 (0.50-0.91)	0.011[1]	0.68 (0.50-0.91)	0.011[1]	0.70 (0.51-0.96)	0.027[1]
Age ≥ 53 years old (n = 81,969)						
None	1.00		1.00		1.00	
1/2 cup each time	1.05 (0.83-1.32)	0.693	0.12 (0.89-1.41)	0.327	1.05 (0.82-1.35)	0.678
1 cup each time	0.82 (0.74-0.92)	<0.001[1]	0.88 (0.79-0.98)	0.025[1]	0.84 (0.73-0.96)	0.010[1]
2 cups each time	0.92 (0.73-1.15)	0.451	1.01 (0.80-1.27)	0.932	0.95 (0.74-1.22)	0.680
Men (n = 55,559)						
None	1.00		1.00		1.00	
1/2 cup each time	1.00 (0.66-1.52)	0.982	1.10 (0.72-1.66)	0.664	1.05 (0.67-1.63)	0.843
1 cup each time	1.02 (0.84-1.24)	0.828	1.19 (0.97-1.45)	0.089	1.10 (0.87-1.39)	0.420
2 cups each time	1.07 (0.78-1.48)	0.668	1.26 (0.91-1.75)	0.166	1.20 (0.85-1.71)	0.301
Women (n = 106,489)						
None	1.00		1.00		1.00	
1/2 cup each time	0.94 (0.77-1.16)	0.592	1.05 (0.85-1.29)	0.649	1.05 (0.84-1.32)	0.645
1 cup each time	0.73 (0.66-0.80)	<0.001[1]	0.79 (0.71-0.87)	<0.001[1]	0.79 (0.70-0.90)	<0.001[1]
2 cups each time	0.64 (0.51-0.80)	<0.001[1]	0.72 (0.58-0.91)	0.005[1]	0.71 (0.56-0.90)	0.005[1]
Nonsmoker (n = 118,185)						
None	1.00		1.00		1.00	
1/2 cup each time	0.92 (0.75-1.13)	0.440	1.02 (0.83-1.25)	0.881	0.99 (0.79-1.23)	0.907
1 cup each time	0.77 (0.70-0.85)	<0.001[1]	0.84 (0.76-0.93)	0.001[1]	0.84 (0.74-0.94)	0.004[1]

2 cups each time	0.68 (0.54-0.84)	<0.001[1]	0.78 (0.63-0.98)	0.031[1]	0.76 (0.60-0.95)	0.019[1]
Past smoker (n = 23,761)						
None	1.00		1.00		1.00	
1/2 cup each time	1.27 (0.76-2.13)	0.368	1.30 (0.77-2.19)	0.324	0.19 (0.68-2.08)	0.534
1 cup each time	1.01 (0.77-1.31)	0.952	1.08 (0.83-1.42)	0.555	0.95 (0.69-1.29)	0.728
2 cups each time	1.23 (0.79-1.89)	0.356	1.29 (0.83-2.00)	0.254	1.14 (0.71-1.83)	0.594
Current smoker (n = 20,102)						
None	1.00		1.00		1.00	
1/2 cup each time	0.91 (0.46-1.80)	0.782	1.04 (0.52-2.09)	0.909	1.38 (0.67-2.84)	0.379
1 cup each time	0.60 (0.42-0.86)	0.006[1]	0.72 (0.50-1.05)	0.089	0.82 (0.54-1.24)	0.345
2 cups each time	0.57 (0.33-0.99)	0.048[1]	0.65 (0.37-1.14)	0.132	0.80 (0.44-1.45)	0.463

[1] Logistic regression model, Significance at $P < 0.05$

[2] Model 1 was adjusted for age, sex, BMI category, income, smoking status, alcohol consumption, and nutritional intake (total calories, protein, fat, and carbohydrate).

[3] Model 2 was adjusted for model 1 plus amount of green tea and soda drink intake.

Table S3. Crude and adjusted odds ratios (95% confidence interval) for asthma by green tea intake (frequency) according to age, sex, and smoking status.

Characteristics	Odds ratios for asthma					
	Crude	P-value	Model 1[2]	P-value	Model 2[3]	P-value
Age < 53 years old (n = 80,079)						
None	1.00		1.00		1.00	
1 time (m) through 6 times (w)	1.03 (0.88-1.21)	0.711	1.04 (0.88-1.23)	0.629	0.98 (0.80-1.20)	0.866
1-2 times (d)	0.81 (0.70-0.94)	0.005[1]	0.82 (0.71-0.95)	0.008[1]	0.93 (0.78-1.12)	0.442
≥ 3 times (d)	0.76 (0.63-0.92)	0.004[1]	0.81 (0.66-0.98)	0.031[1]	0.81 (0.63-1.04)	0.093
Age ≥ 53 years old (n = 81,969)						
None	1.00		1.00		1.00	
1 time (m) through 6 times (w)	1.15 (1.02-1.30)	0.023[1]	1.15 (1.02-1.30)	0.020[1]	1.12 (0.95-1.32)	0.184
1-2 times (d)	0.89 (0.80-0.99)	0.041[1]	0.90 (0.80-1.00)	0.059	1.01 (0.87-1.18)	0.895
≥ 3 times (d)	0.92 (0.78-1.08)	0.307	0.99 (0.83-1.17)	0.896	1.19 (0.92-1.56)	0.187
Men (n = 55,559)						
None	1.00		1.00		1.00	
1 time (m) through 6 times (w)	1.44 (1.21-1.73)	<0.001[1]	1.47 (0.23-1.76)	<0.001[1]	1.34 (1.05-1.71)	0.020[1]
1-2 times (d)	1.02 (0.86-1.21)	0.771	1.04 (0.88-1.23)	0.669	1.07 (0.85-1.34)	0.550
≥ 3 times (d)	0.90 (0.74-1.09)	0.273	1.01 (0.83-1.24)	0.884	0.93 (0.70-1.24)	0.634
Women (n = 106,489)						
None	1.00		1.00		1.00	
1 time (m) through 6 times (w)	1.00 (0.89-1.13)	0.943	1.00 (0.89-1.13)	0.947	0.98 (0.84-1.14)	0.762
1-2 times (d)	0.81 (0.73-0.90)	<0.001[1]	0.82 (0.74-0.91)	<0.001[1]	0.96 (0.84-1.10)	0.578
≥ 3 times (d)	0.85 (0.72-0.99)	0.049[1]	0.90 (0.76-1.07)	0.234	1.06 (0.84-1.34)	0.639
Nonsmoker (n = 118,185)						
None	1.00		1.00		1.00	
1 time (m) through 6 times (w)	1.07 (0.96-1.19)	0.246	1.07 (0.96-1.19)	0.244	1.01 (0.88-1.17)	0.843
1-2 times (d)	0.84 (0.76-0.93)	0.001[1]	0.85 (0.76-0.94)	0.001[1]	0.99 (0.87-1.13)	0.888

≥ 3 times (d)	0.85 (0.73-1.00)	0.055	0.94 (0.80-1.11)	0.462	1.06 (0.84-1.34)	0.595
Past smoker (n = 23,761)						
None	1.00		1.00		1.00	
1 time (m) through 6 times (w)	1.38 (1.08-1.77)	0.011[1]	1.41 (1.10-1.81)	0.008[1]	1.30 (0.93-1.81)	0.125
1-2 times (d)	1.15 (0.92-1.43)	0.208	1.14 (0.91-1.42)	0.259	1.17 (0.87-1.57)	0.287
≥ 3 times (d)	0.98 (0.75-1.29)	0.915	1.04 (0.78-1.37)	0.807	1.16 (0.77-1.77)	0.475
Current smoker (n = 20,102)						
None	1.00		1.00		1.00	
1 time (m) through 6 times (w)	1.00 (0.71-1.42)	0.983	1.08 (0.76-1.54)	0.649	1.26 (0.79-2.00)	0.333
1-2 times (d)	0.63 (0.46-0.85)	0.003[1]	0.67 (0.49-0.91)	0.012[1]	0.71 (0.47-1.05)	0.088
≥ 3 times (d)	0.64 (0.48-0.86)	0.003[1]	0.74 (0.54-1.00)	0.052	0.72 (0.48-1.07)	0.103

[1] Logistic regression model, Significance at $P < 0.05$

[2] Model 1 was adjusted for age, sex, BMI category, income, smoking status, alcohol consumption, and nutritional intake (total calories, protein, fat, and carbohydrate).

[3] Model 2 was adjusted for model 1 plus frequency of coffee and soda drink intake.

Table S4. Crude and adjusted odds ratios (95% confidence interval) for asthma by green tea intake (amount) according to age, sex, and smoking status.

Characteristics	Odds ratios for asthma					
	Crude	P-value	Model 1[2]	P-value	Model 2[3]	P-value
Age < 53 years old (n = 80,079)						
None	1.00		1.00		1.00	
1/2 cup each time	0.82 (0.70-0.97)	0.021[1]	0.85 (0.72-1.00)	0.053	0.87 (0.73-1.04)	0.136
1 cup each time	0.87 (0.76-0.99)	0.036[1]	0.89 (0.78-1.02)	0.097	0.83 (0.80-1.07)	0.318
2 cups each time	0.96 (0.60-1.55)	0.879	0.95 (0.59-1.53)	0.839	1.06 (0.65-1.72)	0.821
Age ≥ 53 years old (n = 81,969)						
None	1.00		1.00		1.00	
1/2 cup each time	1.04 (0.91-1.18)	0.591	1.09 (0.95-1.24)	0.224	1.15 (0.99-1.34)	0.059
1 cup each time	0.95 (0.86-1.04)	0.271	0.96 (0.87-1.06)	0.413	1.05 (0.93-1.18)	0.456
2 cups each time	1.11 (0.79-1.56)	0.554	1.06 (0.75-1.50)	0.732	1.13 (0.79-1.61)	0.497
Men (n = 55,559)						
None	1.00		1.00		1.00	
1/2 cup each time	1.04 (0.86-1.25)	0.667	1.13 (0.94-1.37)	0.200	1.09 (0.89-1.34)	0.410
1 cup each time	1.10 (0.95-1.27)	0.203	1.16 (0.99-1.35)	0.055	1.11 (0.94-1.33)	0.222
2 cups each time	1.12 (0.69-1.81)	0.638	1.08 (0.67-1.75)	0.755	1.03 (0.62-1.68)	0.919
Women (n = 106,489)						
None	1.00		1.00		1.00	
1/2 cup each time	0.89 (0.78-1.01)	0.062	0.94 (0.83-1.07)	0.379	1.02 (0.89-1.17)	0.812
1 cup each time	0.87 (0.79-0.95)	0.003[1]	0.86 (0.78-0.95)	0.003[1]	0.96 (0.86-1.07)	0.479
2 cups each time	1.08 (0.77-1.51)	0.670	1.03 (0.73-1.45)	0.876	1.16 (0.82-1.65)	0.397
Nonsmoker (n = 118,185)						
None	1.00		1.00		1.00	
1/2 cup each time	0.91 (0.81-1.03)	0.127	0.97 (0.86-1.10)	0.655	1.03 (0.90-1.18)	0.654
1 cup each time	0.91 (0.83-0.99)	0.041[1]	0.91 (0.83-1.00)	0.058	0.99 (0.89-1.11)	0.920

2 cups each time	1.01 (0.72-1.42)	0.934	0.98 (0.70-1.38)	0.929	1.08 (0.77-1.54)	0.645
Past smoker (n = 23,761)						
None	1.00		1.00		1.00	
1/2 cup each time	1.14 (0.88-1.46)	0.322	1.20 (0.93-1.54)	0.170	1.20 (0.91-1.60)	0.194
1 cup each time	1.16 (0.95-1.41)	0.154	1.17 (0.95-1.43)	0.142	1.19 (0.94-1.50)	0.151
2 cups each time	1.66 (0.93-2.94)	0.083	1.54 (0.86-2.75)	0.142	1.52 (0.83-2.76)	1.173
Current smoker (n = 20,102)						
None	1.00		1.00		1.00	
1/2 cup each time	0.67 (0.48-0.94)	0.020[1]	0.77 (0.55-1.09)	0.138	0.83 (0.58-1.19)	0.313
1 cup each time	0.71 (0.55-0.91)	0.008[1]	0.78 (0.60-1.02)	0.069	0.83 (0.62-1.12)	0.219
2 cups each time	0.65 (0.26-1.61)	0.355	0.62 (0.25-1.54)	0.300	0.70 (0.27-1.77)	0.448

[1] Logistic regression model, Significance at P < 0.05

[2] Model 1 was adjusted for age, sex, BMI category, income, smoking status, alcohol consumption, and nutritional intake (total calories, protein, fat, and carbohydrate).

[3] Model 2 was adjusted for model 1 plus amount of coffee and soda drink intake.

Table S5. Crude and adjusted odds ratios (95% confidence interval) for asthma by soda drink intake (frequency) according to age, sex, and smoking status.

Characteristics	Odds ratios for asthma					
	Crude	P-value	Model 1[2]	P-value	Model 2[3]	P-value
Age < 53 years old (n = 80,079)						
None	1.00		1.00		1.00	
1 time (m) through 6 times (w)	1.06 (0.9-1.21)	0.351	1.07 (0.94-1.22)	0.288	1.04 (091-1.19)	0.528
1-2 times (d)	0.87 (0.72-1.05)	0.148	0.90 (0.74-109)	0.290	0.92 (0.76-1.12)	0.424
≥ 3 times (d)	0.97 (0.72-1.31)	0.858	1.02 (0.75-1.39)	0.886	1.02 (0.75-1.39)	0.901
Age ≥ 53 years old (n = 81,969)						
None	1.00		1.00		1.00	
1 time (m) through 6 times (w)	1.06 (0.96-1.16)	0.252	1.11 (1.01-1.22)	0.037[1]	1.08 (0.9-1.19)	0.111
1-2 times (d)	0.87 (0.75-1.02)	0.085	0.93 (0.80-1.09)	0.401	0.96 (0.82-1.12)	0.591
≥ 3 times (d)	1.02 (0.78-1.33)	0.874	1.11 (0.85-1.46)	0.447	1.11 (0.85-1.46)	0.430
Men (n = 55,559)						
None	1.00		1.00		1.00	
1 time (m) through 6 times (w)	1.01 (0.87-1.16)	0.920	1.08 (0.93-1.24)	0.304	1.03 (0.89-1.19)	0.721
1-2 times (d)	0.82 (0.66-1.01)	0.060	0.92 (0.74-1.14)	0.425	0.83 (0.75-1.15)	0.499
≥ 3 times (d)	0.91 (0.64-1.27)	0.573	1.04 (0.74-1.47)	0.820	1.05 (0.74-1.48)	0.799
Women (n = 106,489)						
None	1.00		1.00		1.00	
1 time (m) through 6 times (w)	1.02 (0.93-1.12)	0.635	1.10 (1.00-0.21)	0.042[1]	1.08 (0.99-1.19)	0.096
1-2 times (d)	0.83 (0.72-0.96)	0.013[1]	0.92 (0.79-1.06)	0.245	0.95 (0.82-1.10)	0.469
≥ 3 times (d)	0.95 (0.74-1.21)	0.684	1.06 (0.83-1.36)	0.638	1.07 (0.83-1.37)	0.601
Nonsmoker (n = 118,185)						
None	1.00		1.00		1.00	
1 time (m) through 6 times (w)	1.03 (0.94-1.13)	0.472	1.10 (1.01-1.21)	0.030[1]	1.08 (0.99-1.18)	0.098
1-2 times (d)	0.84 (0.73-0.97)	0.017[1]	0.93 (0.81-1.07)	0.317	0.96 (0.83-1.11)	0.574

≥ 3 times (d)	0.98 (0.78-1.24)	0.882	1.11 (0.88-1.41)	0.381	1.11 (0.88-1.41)	0.377
Past smoker (n = 23,761)						
None	1.00		1.00		1.00	
1 time (m) through 6 times (w)	0.96 (0.79-1.16)	0.647	1.06 (0.87-1.28)	0.582	1.02 (0.83-1.24)	0.868
1-2 times (d)	0.77 (0.58-1.03)	0.083	0.88 (0.65-1.18)	0.392	0.88 (0.65-1.18)	0.384
≥ 3 times (d)	0.90 (0.56-1.43)	0.647	1.03 (0.64-1.65)	0.916	1.04 (0.65-1.67)	0.879
Current smoker (n = 20,102)						
None	1.00		1.00		1.00	
1 time (m) through 6 times (w)	0.94 (0.74-1.20)	0.641	1.09 (0.85-1.41)	0.475	1.06 (0.82-1.36)	0.662
1-2 times (d)	0.72 (0.49-1.06)	0.098	0.90 (0.60-1.33)	0.589	0.91 (0.61-1.35)	0.644
≥ 3 times (d)	0.60 (0.29-1.22)	0.157	0.75 (0.36-1.55)	0.438	0.74 (0.36-1.53)	0.414

[1] Logistic regression model, Significance at $P < 0.05$

[2] Model 1 was adjusted for age, sex, BMI category, income, smoking status, alcohol consumption, and nutritional intake (total calories, protein, fat, and carbohydrate).

[3] Model 2 was adjusted for model 1 plus frequency of coffee and green tea intake.

Table S6. Crude and adjusted odds ratios (95% confidence interval) for asthma by soda drink intake (amount) according to age, sex, and smoking status.

Characteristics	Odds ratios for asthma					
	Crude	P-value	Model 1[2]	P-value	Model 2[3]	P-value
Age < 53 years old (n = 80,079)						
None	1.00		1.00		1.00	
1/2 cup each time	0.99 (0.71-1.36)	0.943	0.98 (0.71-1.36)	0.916	0.97 (0.69-1.36)	0.858
1 cup each time	1.01 (0.89-1.14)	0.873	1.03 (0.91-1.17)	0.611	1.04 (0.91-1.18)	0.566
2 cups each time	1.07 (0.78-1.48)	0.660	1.09 (0.79-1.51)	0.598	1.13 (0.82-1.57)	0.448
Age ≥ 53 years old (n = 81,969)						
None	1.00		1.00		1.00	
1/2 cup each time	1.02 (0.79-1.32)	0.861	1.08 (0.84-1.39)	0.548	0.99 (0.76-1.30)	0.968
1 cup each time	1.01 (0.92-1.11)	0.762	1.07 (0.97-1.17)	0.152	1.08 (0.98-1.18)	0.126
2 cups each time	1.06 (0.77-1.47)	0.700	1.18 (085-1.63)	0.326	1.13 (0.82-1.57)	0.447
Men (n = 55,559)						
None	1.00		1.00		1.00	
1/2 cup each time	0.94 (0.64-1.39)	0.942	0.98 (0.66-1.44)	0.918	0.99 (0.66-1.48)	0.962
1 cup each time	0.97 (0.84-1.10)	0.966	1.05 (0.92-1.20)	0.472	1.05 (0.92-1.21)	0.462
2 cups each time	0.72 (0.44-1.18)	0.722	0.84 (0.51-1.38)	0.497	0.84 (0.51-1.38)	0.487
Women (n = 106,489)						
None	1.00		1.00		1.00	
1/2 cup each time	0.97 (0.77-1.23)	0.811	1.06 (0.84-1.34)	0.596	0.98 (0.77-1.25)	0.884
1 cup each time	0.97 (0.89-1.06)	0.566	1.05 (0.96-1.15)	0.238	1.06 (0.97-1.16)	0.180
2 cups each time	1.05 (0.82-1.36)	0.697	1.22 (0.94-1.58)	0.129	1.23 (0.95-1.60)	0.116
Nonsmoker (n = 118,185)						
None	1.00		1.00		1.00	
1/2 cup each time	1.04 (0.84-1.30)	0.710	1.13 (0.90-1.40)	0.288	1.08 (0.86-1.35)	0.526
1 cup each time	0.98 (0.90-1.07)	0.660	1.06 (0.97-1.15)	0.203	1.06 (0.97-1.16)	0.164

2 cups each time	1.09 (0.85-1.39)	0.502	1.27 (0.99-1.64)	0.058	1.29 (1.00-1.67)	0.047[1]
Past smoker (n = 23,761)						
None	1.00		1.00		1.00	
1/2 cup each time	0.89 (0.52-1.51)	0.662	0.96 (0.57-1.64)	0.888	0.90 (0.52-1.56)	0.706
1 cup each time	0.92 (0.77-1.10)	0.378	1.03 (0.85-1.24)	0.765	1.03 (0.86-1.25)	0.726
2 cups each time	0.65 (0.33-1.28)	0.216	0.75 (0.38-1.47)	0.400	0.71 (0.36-1.41)	0.330
Current smoker (n = 20,102)						
None	1.00		1.00		1.00	
1/2 cup each time	0.25 (0.06-1.01)	0.051	0.27 (0.07-1.10)	0.068	0.24 (0.06-0.98)	0.047[1]
1 cup each time	0.91 (0.73-1.15)	0.432	1.09 (0.86-1.38)	0.457	1.09 (0.86-1.38)	0.478
2 cups each time	0.49 (0.18-1.33)	0.161	0.58 (0.21-1.59)	0.291	0.58 (0.21-1.60)	0.294

[1] Logistic regression model, Significance at $P < 0.05$

[2] Model 1 was adjusted for age, sex, BMI category, income, smoking status, alcohol consumption, and nutritional intake (total calories, protein, fat, and carbohydrate).

[3] Model 2 was adjusted for model 1 plus amount of coffee and green tea intake.

Table S7. Frequency of coffee/green tea/soda drink intake according to sex

Characteristics	Total participants		P-value
	Men	Women	
Frequency of coffee			<0.001[1]
None	7,136 (12.8)	19,971 (18.8)	
1 time (m) through 6 times (w)	10,659 (19.2)	23,919 (22.5)	
1-2 times (d)	21,678 (39.0)	46,796 (43.9)	
≥ 3 times (d)	16,086 (29.0)	15,803 (14.8)	
Amount of coffee			<0.001[1]
None	7,136 (12.8)	19,971 (18.8)	
1/2 cup each time	1,671 (3.0)	4,589 (4.3)	
1 cup each time	43,675 (78.6)	76,422 (71.8)	
2 cups each time	3,077 (5.5)	5,507 (5.2)	
Frequency of green tea			<0.001[1]
None	19,186 (34.5)	50,406 (47.3)	
1 time (m) through 6 times (w)	8,629 (15.5)	17,576 (16.5)	
1-2 times (d)	16,071 (28.9)	29,801 (28.0)	
≥ 3 times (d)	11,673 (21.0)	8,706 (8.2)	
Amount of green tea			<0.001[1]
None	19,186 (34.5)	50,406 (47.3)	
1/2 cup each time	10,463 (18.8)	16,713 (15.7)	
1 cup each time	24,915 (44.8)	37,893 (35.6)	
2 cups each time	995 (1.8)	1,477 (1.4)	
Frequency of soda drink			<0.001[1]
None	22,005 (39.6)	43,245 (40.6)	
1 time (m) through 6 times (w)	23,212 (41.8)	46,295 (43.5)	
1-2 times (d)	8,014 (14.4)	13,433 (12.6)	
≥ 3 times (d)	2,328 (4.2)	3,516 (3.3)	
Amount of soda drink			<0.001[1]
None	22,005 (39.6)	43,245 (40.6)	
1/2 cup each time	1,698 (3.1)	3,881 (3.6)	
1 cup each time	30,517 (54.9)	56,407 (53.0)	
2 cups each time	1,339 (2.4)	2,956 (2.8)	

[1] Independent T-test or Chi-square test. Significance at $P < 0.05$

d= day; w = week; m = month

Table S8. Incidence of developed asthma during the follow-up period according to the frequency and amount of coffee/green tea/soda drink intake

Characteristics	Asthma incidence (n, %)	P-value
Frequency of coffee		<0.001[1]
None	59 (16.0)	
1 time (m) through 6 times (w)	96 (26.0)	
1-2 times (d)	153 (41.5)	
≥ 3 times (d)	61 (16.5)	
Amount of coffee		<0.001[1]
None	59 (16.0)	
1/2 cup each time	18 (4.9)	
1 cup each time	277 (75.1)	
2 cups each time	15 (4.1)	
Frequency of green tea		<0.001[1]
None	154 (41.7)	
1 time (m) through 6 times (w)	78 (21.1)	
1-2 times (d)	101 (27.4)	
≥ 3 times (d)	36 (9.8)	
Amount of green tea		<0.001[1]
None	154 (41.7)	
1/2 cup each time	55 (14.9)	
1 cup each time	154 (41.7)	
2 cups each time	6 (1.6)	
Frequency of soda drink		<0.001[1]
None	143 (38.8)	
1 time (m) through 6 times (w)	178 (48.2)	
1-2 times (d)	33 (8.9)	
≥ 3 times (d)	15 (4.1)	
Amount of soda drink		<0.001[1]
None	143 (38.8)	
1/2 cup each time	12 (3.3)	
1 cup each time	204 (55.3)	
2 cups each time	10 (2.7)	

[1] Chi-square test. Significance at $P < 0.05$

d= day; w = week; m = month

Table S9. Adjusted odds ratios (95% confidence interval) for asthma by coffee, green tea, and soda drink intake

Characteristics	Odds ratio for asthma	P-value
Frequency of coffee		
None	1.00	
1 time (m) through 6 times (w)	0.99 (0.87-1.14)	0.936
1-2 times (d)	0.84 (0.75-0.96)	0.008[1]
≥ 3 times (d)	0.89 (0.75-1.05)	0.172
Amount of coffee		
None	1.00	
1/2 cup each time	1.07 (0.88-1.31)	0.491
1 cup each time	0.89 (0.80-0.99)	0.030[1]
2 cups each time	0.86 (0.71-1.05)	0.132
Frequency of green tea		
None	1.00	
1 time (m) through 6 times (w)	1.07 (0.94-1.21)	0.311
1-2 times (d)	0.99 (0.88-1.11)	0.904
≥ 3 times (d)	0.99 (0.83-1.19)	0.975
Amount of green tea		
None	1.00	
1/2 cup each time	1.03 (0.92-1.16)	0.559
1 cup each time	1.01 (0.92-1.11)	0.831
2 cups each time	1.13 (0.85-1.50)	0.394
Frequency of soda drink		
None	1.00	
1 time (m) through 6 times (w)	1.07 (0.99-1.15)	0.107
1-2 times (d)	0.94 (0.83-1.06)	0.322
≥ 3 times (d)	1.06 (0.87-1.30)	0.569
Amount of soda drink		
None	1.00	
1/2 cup each time	0.97 (0.79-1.19)	0.780
1 cup each time	1.06 (0.98-1.14)	0.151
2 cups each time	1.13 (0.90-1.42)	0.298

[1] Logistic regression model adjusted for age, sex, income, smoking status, alcohol consumption, and total calories. Significance at $P < 0.05$

d= day; w = week; m = month
